# The Hippo Signaling Pathway in Cardiac Development and Diseases

**DOI:** 10.3389/fcell.2019.00211

**Published:** 2019-10-01

**Authors:** Masum M. Mia, Manvendra K. Singh

**Affiliations:** ^1^Program in Cardiovascular and Metabolic Disorders, Duke-NUS Medical School, Singapore, Singapore; ^2^National Heart Research Institute Singapore, National Heart Center, Singapore, Singapore

**Keywords:** hippo signaling, cardiac development, hypertrophy, ischemia – reperfusion, cardiomyoapthies

## Abstract

Heart disease continues to be the leading cause of morbidity and mortality worldwide. Cardiac malformation during development could lead to embryonic or postnatal death. However, matured heart tissue has a very limited regenerative capacity. Thus, loss of cardiomyocytes from injury or diseases in adults could lead to heart failure. The Hippo signaling pathway is a newly identified signaling cascade that modulates regenerative response by regulating cardiomyocyte proliferation in the embryonic heart, as well as in postnatal hearts after injury. In this review, we summarize recent findings highlighting the function and regulation of the Hippo signaling pathway in cardiac development and diseases.

## Introduction

**FIGURE 1:**
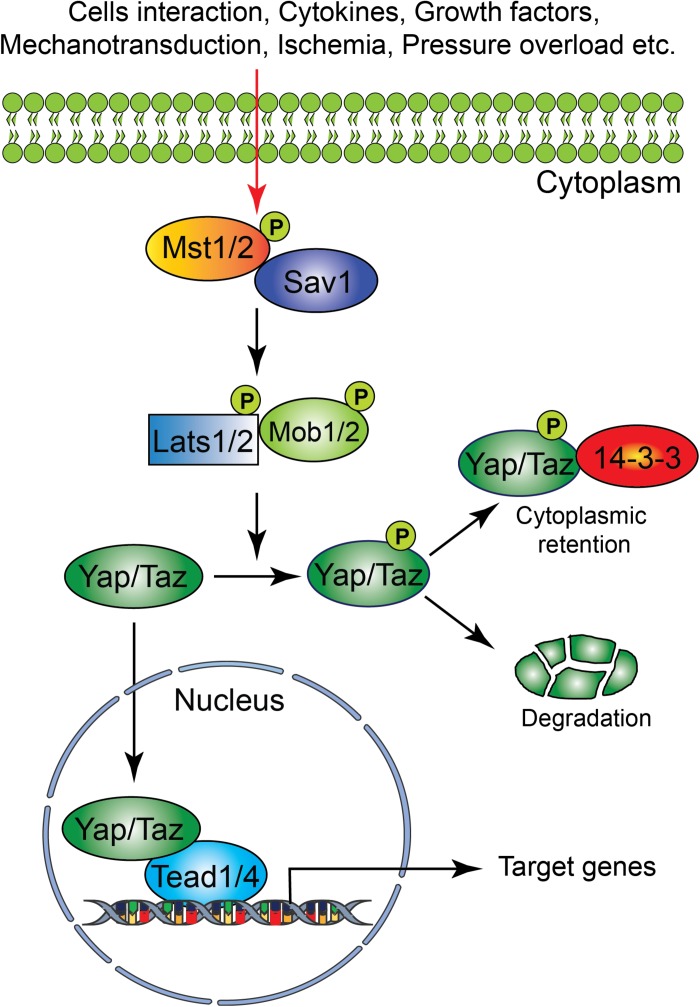
Schematic representation of the core components of the Hippo signaling pathway. Several physiological and pathological signals can activate the Hippo signaling pathway in the heart. The physiological signal includes cell–cell interaction, cytokines, and growth factors, whereas the pathological signal includes oxidative stress (ischemia-reperfusion injury), mechanical stress (pressure overload) and injury (myocardial infarction). The core Hippo signaling pathway consists of serine/threonine kinases, transcriptional coactivators, and transcription factors. Upon activation, the upstream kinases (Mst1/2, Lats1/2, Sav1, and MOB1) promote phosphorylation of downstream mediators Yap and Taz, resulting in their cytoplasmic retention or degradation. In contrast, inactivation of upstream kinases leads to nuclear translocation of Yap and Taz, where they bind to various transcription factors including Tead factors (Tead1-4) and regulate target gene expression.

The Hippo signaling pathway components were first identified and characterized in *Drosophila*. However, the core components of the Hippo signaling pathway are highly conserved in mammals. The Hippo signaling pathway is a vital pathway that controls the development and homeostasis of many mammalian organs including heart by regulating cell proliferation, apoptosis, cell fate decisions, and stem cell self-renewal (Halder and Johnson, 2011; Zhao et al., 2011; Zhou et al., 2015). The mammalian core components of the Hippo signaling pathway are composed of a serine/threonine kinase cascade, transcriptional coactivators and transcription factors (Figure 1). Upon activation of a kinase cascade, the sterile 20-like protein kinase (Mst1/2) interacts with an adaptor protein Salvador (*Salv*) and phosphorylates many proteins including Salv, large tumor suppressor (Lats1/2) and Lats1/2-interacting proteins MOB1. This leads to phosphorylation, cytoplasmic retention, and degradation of the transcriptional coactivators, Yes-associated protein (Yap) and transcriptional coactivator with PDZ-binding motif (Taz, also known as Wwtr1). In contrast, inactivation of upstream kinases leads to nuclear translocation of both Yap and Taz, where they further interact with various transcription factors including TEA domain family member (Tead1-4). This results in regulation of transcriptional activity for downstream target genes involved in cell proliferation, survival and differentiation (Halder and Johnson, 2011; Heallen et al., 2011; Zhao et al., 2011; Zhou et al., 2015).

## The Hippo Signaling Pathway in Cardiac Development

**FIGURE 2:**
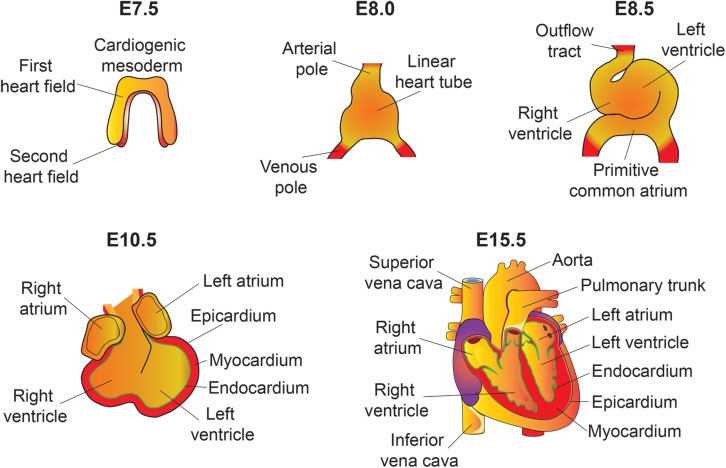
An overview of mouse heart development. Heart development begins with the specification of cardiogenic mesoderm cells in the primitive streak at E6.5. At E7.5, these mesodermal precursors migrate away from the primitive streak to form a bow-shaped structure called the cardiac crescent. Cardiac crescent can be divided into two major cardiac progenitor pools: the first and second heart field. The cardiac progenitor cells from the first heart field contribute to the linear heart tube, whereas the second heart field contribute to portions of the atria, the outflow tract, and the right ventricle. As embryonic development proceeds, the progenitor cells fuse at midline and form a primitive linear heart tube. At E8.5, the linear heart tube undergoes looping leading to formation of the outflow tract, primitive ventricles, and atria. At early stages, the heart consists of two layers: an inner endocardium and an outer myocardium. Between E9 and10.5, progenitor cells from different sources (including neural crest and proepicardial organ) migrate and contribute to the outflow tract and ventricular chambers. Myocardial layer expands and forms compact and trabecular myocardium. The proepicardial progenitor cells migrate over the heart surface and form epicardium. Epicardial-derived cells contribute to the formation of the coronary vasculature. Heart maturation involves a series of septation events and valve formation that results in a fully functional four-chambered heart integrated with the circulatory system by E15.5.

**FIGURE 3:**
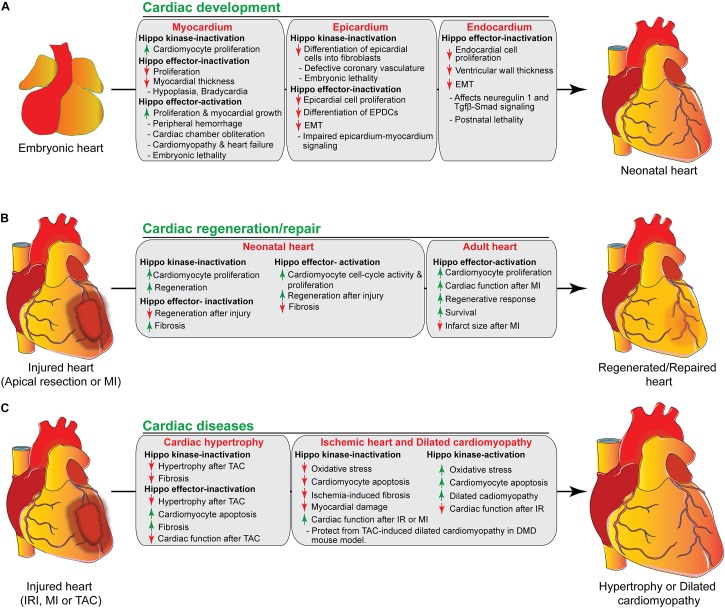
The role of Hippo signaling components in cardiac development, regeneration/repair, and diseases. **(A)** During cardiac development, activation/inactivation of the Hippo signaling components modulates proliferation and differentiation of cardiomyocytes, epicardial and endocardial cells, resulting in defective cardiogenesis and embryonic lethality as shown in the figure. **(B)** Activation/inactivation of the Hippo signaling components differently regulates the regenerative response of neonatal and adult hearts after injury. **(C)** Activation/inactivation of the Hippo signaling components leads to many cardiac diseases as described in the figure. EPDCs, epicardium-derived cells; EMT, endothelial mesenchymal transition; MI, myocardial infarction; IRI, ischemia/reperfusion injury, TAC, transverse aortic constriction; DMD, Duchenne Muscular Dystrophy.

The heart is the first functional organ formed during embryonic development. It pumps blood filled with oxygen and nutrients through the blood vessels to the tissues, required for further organogenesis. To maintain its function and carry out its physiologic cardiac output, the heart needs to develop properly. A smaller size heart is not able to eject sufficient blood into the circulation system, resulting in cardiac outflow disruption that leads to cardiomyopathies (diseases of the myocardium, or heart muscle affecting the pumping ability of the heart) (Heallen et al., 2011; Zhao et al., 2011). Deregulation of the Hippo signaling pathway leads to various congenital cardiac abnormalities. Hippo signaling components are expressed in the cells of all three cardiac layers (myocardium, epicardium, and endocardium), and play an important role during cardiac development (Figures 2, 3).

## Hippo Signaling Pathway in the Myocardium

Cardiomyocyte proliferation is vital for proper myocardial growth during heart development. A number of studies using either loss or gain of function genetic experiments have demonstrated that Hippo signaling pathway plays a central role in regulating cardiomyocyte proliferation during heart development to maintain a normal mammalian heart size (Tables 1, 2) (Heallen et al., 2011; Xin et al., 2011; von Gise et al., 2012). To investigate the role of Hippo pathway in cardiac development, Heallen et al. (2011) used gene inactivation approach to delete *Sav1*, *Mst1/2*, and *Lats2* in the embryonic mouse heart by crossing their conditional-flox alleles with *Nkx2.5^Cre^* line. They found that cardiac-specific deletion of *Sav1*, *Mst1/2* or *Lats2* results in elevated cardiomyocyte proliferation, trabecular expansion, and thickening of compact ventricular myocardium leading to cardiomegaly (abnormal enlargement of the heart) without altering the cell size. However, the overall patterning of the heart was preserved in these mutants (Heallen et al., 2011). Further studies were also conducted to examine the effect of Yap’s gain and loss of function on cardiac development (Xin et al., 2011; von Gise et al., 2012). Cardiomyocyte specific deletion of *Yap* using *Nkx2.5^Cre^* or *Tnnt2*^Cre^ driver resulted in impaired cardiomyocyte proliferation that led to lethal cardiac hypoplasia and severely thinned myocardial layers (Xin et al., 2011; von Gise et al., 2012). Conversely, cardiomyocyte-specific overexpression of activated Yap (Yap^S112A^) resulted in enhanced cardiomyocyte proliferation leading to cardiac overgrowth in neonatal mice (Xin et al., 2011). Similarly, inducible expression of activated Yap (Yap^S127A^) using *Tnnt2*^Cre^ line showed peripheral hemorrhage, cardiomegaly and dramatic myocardial overgrowth causing cardiac chamber obliteration, all of which resulted in embryonic death by E15.5 (von Gise et al., 2012). Consistent with these findings, Monroe et al. demonstrated that overexpression of activated Yap in adult cardiomyocytes (*αMHC^Cre–ERT2^;Yap^5SA^*) leads to increased proliferation, resulting in thickened ventricular walls and improved cardiac function (Monroe et al., 2019). Despite the improved cardiac function, these mice die within 4 days after the last dose of tamoxifen, given to induce the cre activity in cardiomyocytes. Furthermore, Monroe et al. demonstrated that Yap^5SA^ overexpression induced adult cardiomyocytes into a fetal cell state by increasing the accessibility of embryonic cardiac enhancers in the adult heart (Monroe et al., 2019). In contrast to the well-established role of Yap in the heart, the role of Taz has not been studied extensively. Recently, Xin et al. (2013) demonstrated that cardiac-specific *Taz* knockout mice are viable and do not show any obvious cardiac defects. However, various combinations of *Taz* and *Yap* deletions in cardiomyocytes resulted in gene dosage-dependent cardiac defects. The cardiac defects in *Yap/Taz* mutants hearts were also associated with an increase in cardiomyocyte proliferation and a decrease in apoptosis (Xin et al., 2013). Furthermore, Murakami et al. (2005) revealed that the Hippo effector Taz physically interacts and modulates TBX5 transcriptional activity required for cardiomyogenesis. The Tead family transcription factors interact with both Yap and Taz via the N-terminal Tead binding domains in Yap/Taz. Disruption of *Tead1* locus by a retroviral gene trapping resulted in formation of the thinned ventricular wall, enlarged pericardial cavity, bradycardia and embryonic lethality (Chen et al., 1994). Importantly, overexpression of Tead1 in the mouse heart induced cardiomyopathy and heart failure, suggesting that Tead1 is vital for proper heart development (Tsika et al., 2010).

**TABLE 1:** Hippo signaling kinases in cardiac development and disease.

**Genes**	**Model**	**Cardiac phenotypes**	**References**
*Mst1/2*	*Nkx2.5^Cre/+^;Mst1^f/f^; Mst2^f/f^*	Cardiomegaly due to increased cardiomyocyte proliferation.	Heallen et al., 2011
	*Mst1^–/–^; Mst2^–/–^*	Developmental defects, Embryonic death between E9.5–E10.5.	Oh et al., 2009; Song et al., 2010
	*CAGG*^Cre–ER^; Mst1^–/–^; Mst2^f/–^	Enlarged heart (partial penetrance).	Song et al., 2010
	α*MHC-Mst1 Tg*	Premature death (∼P15) due to heart failure, increased cardiomyocyte apoptosis, fibrosis and dilated cardiomyopathy.	Yamamoto et al., 2003
	α*MHC-Mst1^K59R^ Tg*	No premature death, no sign of heart failure, reduced apoptosis after I/R injury, reduced apoptosis and fibrosis, no effect on cardiac hypertrophy.	Yamamoto et al., 2003; Odashima et al., 2007
	*Mst2^–/–^*	No cardiac defects at the basal condition. Reduction of hypertrophy and fibrosis in response to pressure overload.	Oh et al., 2009; Zi et al., 2014
	*Mst1^–/–^*	No cardiac defects at the basal condition.	Oh et al., 2009
	α*-MHC^MerCreMer^:Mst1^f/+^*	Decreased Angiotensin II-induced cardiomyocyte apoptosis.	Cheng et al., 2019
*Lats1/2*	*Lats2^–/–^*	Ventricular hypoplasia (partial penetrance). Embryonic lethality at E10.5.	McPherson et al., 2004
	*Nkx2.5^Cre/+^;Lats2^f/f^*	Cardiomegaly due to increased cardiomyocyte proliferation.	Heallen et al., 2011
	α*-MHC-Lats2 Tg*	Reduction in the size of the left and right ventricles, reduced left ventricular systolic and diastolic function without affecting baseline apoptosis.	Matsui et al., 2008
	α*-MHC-Lats2^K697A^ Tg*	Ventricular hypertrophy, reduced cardiac myocyte apoptosis induced by TAC.	Matsui et al., 2008
	*Myh6^CreERT2^;Lats1^f/f^;Lats2^f/f^*	Increased cardiomyocyte proliferation, improved regeneration after apical resection.	Heallen et al., 2013
	*Wt1^CreERT2^;Lats1^f/f^;Lats2^f/f^*	Embryonic lethality, defective coronary vasculature remodeling, and impaired differentiation into fibroblasts.	Xiao et al., 2018
*Sav1*	*Nkx2.5^Cre/+^;Salv^f/f^*	Cardiomegaly due to increased cardiomyocyte proliferation.	Heallen et al., 2011
	*Myh6^CreERT2^;Salv^f/f^*	Increased cardiomyocyte renewal, increased cardiomyocyte proliferation, improved regeneration after apical resection or MI.	Heallen et al., 2013

**TABLE 2:** Hippo signaling mediators in cardiac development and disease.

**Genes**	**Model**	**Cardiac phenotypes**	**References**
*Yap, Taz*	*Nkx2.5^Cre/+^;Yap^f/f^*	Decreased cardiomyocyte proliferation, thin ventricle wall. Embryonic death at E10.5.	Xin et al., 2011
	*SM22*α*^Cre/+^;Yap^f/f^*	Ventricular hypoplasia, thin ventricle wall, VSD. Perinatal lethality.	Wang Y. et al., 2014
	*Tnnt2^Cre/+^;Yap^f/f^*	Hypoplastic ventricles, decreased cardiomyocyte proliferation. Embryonic death at E16.5.	von Gise et al., 2012
	α*-MHC^Cre/+^;Yap^f/f^ or*α*-MHC^Cre/+^;Yap^f/+^*	Dilated cardiomyopathy and premature death, increased fibrosis and apoptosis; decreased proliferation, decreased cardiomyocyte proliferation and hypertrophy after MI, impaired cardiac regeneration. Attenuated cardiac hypertrophy, increased apoptosis and fibrosis after pressure overload.	Del Re et al., 2013; Xin et al., 2013; Byun et al., 2019
	α*-MHC^Cre/+^;Yap^S112A^*	Increased cardiomyocyte proliferation, thick myocardium, improved cardiac regeneration after MI.	Xin et al., 2013
	α*-MyHC^Cre–ERT2^;Yap^5SA^*	Increased cardiomyocyte proliferation, thick ventricular wall, and small chambers, and cardiomyocyte hyperplasia. Death within 4 days after tamoxifen treatment.	Monroe et al., 2019
	*β-MHC^Cre/+^;Yap^S112A^*	Increased cardiomyocyte proliferation and thick myocardium.	Xin et al., 2011
	*Tnnt2^Cre/+^;Rosa26^fs–rtTA^;TetO-Yap^S127A^*	Increased cardiomyocyte proliferation, thickened myocardium, cardiomegaly, no hypertrophy.	von Gise et al., 2012
	*Tnnt2^Cre/+^;Yap^f/S79A^*	Cardiac hypoplasia was similar to Yap cardiomyocyte-specific conditional knockout.	von Gise et al., 2012
	*Tie2^Cre/+^;Yap^f/f^*	Hypocellular endocardial cushions due to impaired EMT and reduced endocardial cell proliferation.	Zhang et al., 2014
	α*-MHC^Cre/+^;Taz^f/f^*	No cardiac defects. It enhances cardiac phenotype (decreased proliferation, increased apoptosis, dilated cardiomyopathy, and heart failure) on a Yap null background.	Xin et al., 2013
	*Sema3d^GFPCre/+^;Yap^f/f^;Taz^f/f^*	Impaired epicardial cell proliferation, EMT and differentiation. Embryonic lethality between E11.5–12.5.	Singh et al., 2016
	*Wt1^CreERT2/+^;Yap^f/f^;Taz^f/f^*	Coronary vasculature defects due to impaired epicardial cell proliferation, EMT, and fate determination. Increased post-MI pericardial inflammation and myocardial fibrosis resulting in cardiomyopathy and death.	Singh et al., 2016; Ramjee et al., 2017
	*Nfatc1^IRES–Cre/+^;Yap^f/f^;Taz^f/f^*	Impaired myocardial growth causing early post-natal lethality.	Artap et al., 2018
*Tead*	*Tead1^–/–^*	Enlarged pericardial cavity, bradycardia, thin ventricular wall, reduced number of trabeculae. Embryonic lethality by E11.5.	Chen et al., 1994
	*MCK-Tead1 Tg*	Age-dependent cardiac dysfunction (decreased cardiac output, stroke volume, ejection fraction, and fractional shortening). Misalignment of cardiomyocytes, septal wall thickening, and fibrosis. Heart failure leading to death within 4-days after induction of pressure overload.	Tsika et al., 2010

Multiple signaling pathways including Wnt-β-catenin, insulin-like growth factor (IGF), phosphoinositide 3-kinase (PI3K)–RACα, serine/threonine-protein kinase (AKT) and mammalian Target of Rapamycin (mTOR) have been reported to interact with the Hippo pathway to control cell growth and proliferation during cardiac development (Heallen et al., 2011; Xin et al., 2011). Wnt-β-catenin plays a crucial role in Hippo-Yap-mediated cardiac overgrowth. A recent study using cardiac-specific *Salv* knockout mice demonstrated an inhibitory role of Hippo signaling in the Wnt-β-catenin signaling pathway-dependent cardiac development. Transcriptional profiling of embryonic hearts from *Salv* knockout mice displayed elevated expression of canonical Wnt-β-catenin target genes required for heart development and those with anti-apoptosis function, including *Sox2*, *Snai2*, *Birc2* and *Birc5* (Heallen et al., 2011). Similarly, β-catenin nuclear localization was increased in the cardiomyocytes isolated from *Salv* knockout embryos (Heallen et al., 2011). Furthermore, biochemical studies showed that Yap-Tead forms complexes with β-catenin on the promoter region of *Sox2* and *Snai2* genes (Heallen et al., 2011). Collectively, these results suggest that Hippo signaling inhibit the interaction between Yap and β-catenin in the developing hearts to promote transcription of growth-related genes.

Another signaling pathway that has been demonstrated to cross-talk with Hippo is IGF pathway (Xin et al., 2011). Xin et al. (2011) generated mice overexpressing a constitutively active form of Yap (Yap^S112A^) in the heart. Gene expression profiling of hearts expressing Yap^S112A^ revealed that Yap induces IGF signaling pathway genes such as *Igf1*, *Igfbp2*, and *Igfbp3* (Xin et al., 2011). In addition to IGF pathway genes, Yap also induced β*-catenin (Ctnnb1)* and its downstream target genes including *Kcne3*, *Ndrl*, and *Ier3*, suggesting that β-catenin expression is stabilized in cardiomyocytes (Xin et al., 2011). Furthermore, increased PI3K and phosphorylated AKT levels were observed in Yap^S112A^ expressing cardiomyocytes, indicating increased IGF pathway activity (Xin et al., 2011). Enhanced IGF-AKT results in phosphorylation and inactivation of the β-catenin destruction complex component glycogen synthase kinase 3β (GSK3β), leading to β-catenin stabilization. Moreover, IGF inhibition decreases both Yap activity and β-catenin expression in cardiomyocyte. These suggest that activated Yap increases IGF signaling and stabilizes β-catenin by decreasing GSK3β phosphorylation (Xin et al., 2011). Collectively, they suggest that the Hippo-Yap pathway acts through cardiomyocytes to regulate myocardial growth.

## Hippo Signaling Pathway in the Epicardium

The epicardium is the outermost mesothelial cell layer of the heart. It is essential for proper formation of the coronary vasculature. A number of studies have demonstrated that epicardium-derived cells (EPDCs) can differentiate into vascular smooth muscle cells, coronary endothelial cells and interstitial fibroblasts in the developing heart (Mikawa and Fischman, 1992; Gittenberger-de Groot et al., 1998; Lie-Venema et al., 2007; Red-Horse et al., 2010; Singh et al., 2011; Katz et al., 2012; Singh and Epstein, 2012; von Gise and Pu, 2012; Degenhardt et al., 2013; Epstein et al., 2015; Ramesh et al., 2016). The epicardium is also an important source of growth signals required for proliferation and differentiation of the underlying myocardial cells (Chen et al., 2002; Stuckmann et al., 2003; Lavine et al., 2005; Merki et al., 2005; Pennisi and Mikawa, 2009; Brade et al., 2011; Li et al., 2011; Zhou et al., 2011; Tian and Morrisey, 2012). Similarly, signals from the myocardium can reciprocally modulate epicardial cell behavior (Morabito et al., 2001; Chen et al., 2002; Smart et al., 2007; Mellgren et al., 2008; Bock-Marquette et al., 2009; Smith et al., 2011; Vega-Hernandez et al., 2011). To determine the role of the Hippo signaling pathway in coronary vessel development, Singh et al. (2016) generated an epicardial-specific *Yap/Taz* double mutant embryos using *Sema3d*^GFPCre/+^ and *Wt1^CreERT2/+^* line. Consistent with the observed thin and fragmented myocardium and hemorrhage, the *Yap/Taz* double mutants die around E11.5-E12.5 from cardiovascular insufficiency. Epicardium-myocardium signaling was impaired due to *Yap/Taz* deletion in the epicardium. Inducible deletion of *Yap/Taz* demonstrated that epicardial cell proliferation, epithelial-to-mesenchymal transition, and EPDCs differentiation into cardiac lineages were affected in the double mutant embryos (Singh et al., 2016). In line with our findings, Xiao et al. (2018) demonstrated that the Hippo pathway kinases Lats1 and 2 play an essential role in epicardium development. Epicardial-specific deletion of *Lats1* and *2* resulted in embryonic lethality by E15.5, and defected coronary vasculature remodeling in the double mutant embryos (Xiao et al., 2018). Using single-cell RNA sequencing approach, they showed that *Lats1/2* deficiency in epicardial cells inhibited their differentiation into fibroblasts and remained at intermediate cell state with both epicardial and fibroblast-like features. These results suggest that Lats1/2 activity is required for EPDC progression from an epicardium-derived progenitor to fully differentiated cardiac fibroblasts (Xiao et al., 2018). Both genetic and pharmacological studies further revealed that fibroblasts differentiation is limited by Yap inhibition. Subsequently, this promotes matricellular factor dipeptidyl peptidase-4 expression that modulates the characteristics of extracellular matrix composition required for vascular remodeling during cardiac development (Xiao et al., 2018). Altogether, these findings suggest that the Hippo signaling pathway in the epicardium is necessary for cardiac fibroblast differentiation and coronary vessel development (Singh et al., 2016; Xiao et al., 2018).

## Hippo Signaling Pathway in the Endocardium

Recently, Artap et al. (2018) reported that endocardial Hippo signaling is essential for embryonic heart myocardial growth (Artap et al., 2018). The myocardium’s growth and maturation are partly orchestrated by signals from the neighboring endocardium (Rentschler et al., 2002; Brutsaert, 2003; Sandireddy et al., 2019). Endothelial cells have been shown to express growth factors and cell surface ligands required for cardiac chamber development and maturation. For example, endocardial cells secrete neuregulin 1 (Nrg1) and express the cell surface ligand ephrin B2 (Efnb2), both of which signal to the adjacent myocardial cells to promote myocardium growth and maturation (Gassmann et al., 1995; Lee et al., 1995; Meyer and Birchmeier, 1995). Artap et al. (2018) demonstrated that both Yap and Taz are expressed in both myocardial and endocardial cells of the developing heart. Endocardial-specific deletion of *Yap* and *Taz* using *Nfatc1*^IRES–Cre/+^ allele resulted in postnatal lethality (Artap et al., 2018). Ventricular wall thickness was also reduced in the double mutant embryos. They further showed that *Yap/Taz* deficiency in the endocardium led to reduced expression of Nrg1, an essential endocardial secreted factor that orchestrates the myocardium differentiation/phenotype. Interestingly, exogenous addition of recombinant Nrg1 partly rescued the myocardial phenotype of the mutant hearts. Moreover, they showed that Yap can bind to the promoter regions of *Nrg1*, indicating Yap-mediated regulation of *Nrg1* in the endocardium is necessary for proper myocardial development (Artap et al., 2018). Similarly, conditional deletion of *Yap* in endothelial cells resulted in impaired endothelial mesenchymal transition (EMT) and reduced endocardial cell proliferation, leading to hypocellular endocardial cushions and embryonic lethality (Zhang et al., 2014). Zhang et al. (2014) further demonstrated that the interaction between Hippo signaling mediator Yap and TGFβ-smad (smad2/3/4) pathway is essential for proper atrioventricular cushion development. Loss of Yap function in the endothelial cells also disrupted the Tgfb-smad signaling, resulting in impaired activation of EMT genes such as *Snail1*, *Twist1*, and *Slug* (Zhang et al., 2014). Taken together, these results suggest that Hippo signaling pathway is required for the endocardial/endothelial cells to regulate normal heart development.

## The Hippo Signaling Pathway in Cardiac Regeneration

Since Hippo signaling is reported as an important regulator of cardiomyocyte proliferation during embryonic development (Heallen et al., 2011; Xin et al., 2011; von Gise et al., 2012), it could potentially play a significant role in heart regeneration/repair. In contrast to the adult heart’s limited regenerative capacity, the neonatal mouse heart is capable to regenerate from myocardial injury. However, this capacity is lost by day seven postnatally (P7) (Porrello et al., 2011). Several studies have demonstrated that Hippo signaling pathway is also essential for cardiomyocyte proliferation in postnatal hearts and Yap activation can prolong the 1-week regenerative window. For example, Yap overexpression extends neonatal cardiomyocyte cell cycle activity and promotes regeneration (von Gise et al., 2012). Xin et al. (2013) demonstrated that cardiomyocyte-specific deletion of *Yap* impedes neonatal heart regeneration. Hearts from P2 neonatal wildtype mice were able to fully regenerate after myocardial injury, as evident by minimal fibrosis and healthy myocardial tissue distal to the ligature. In contrast, *Yap* mutant P2 hearts displayed extensive cardiac fibrosis after injury, suggesting impaired regenerative responses (Xin et al., 2013). To demonstrate that Yap activation is sufficient for postnatal heart regeneration, Xin et al. (2013) generated α*MHC-Yap^S112A^* transgenic mice overexpressing activated Yap in the heart under the control of αMHC promoter. Both wildtype and α*MHC-Yap^S112A^* transgenic mice were subjected to myocardial injuries at P7 and analyzed at P21. Wildtype mice showed extensive scar formation, loss of myocardial tissue, and ventricular dilation in their hearts after injury. In contrast, α*MHC-Yap^S112A^* hearts were fully regenerated with minimal or no fibrosis (Xin et al., 2013). Consistent with these results, Heallen et al. (2013) showed that cardiomyocyte-specific inducible inactivation of *Sav1* and *Lats1/2* also increased cardiomyocyte proliferation in the neonatal heart and extended the neonatal regenerative window (Tables 1, 2).

Stimulating cellular proliferation is more challenging in adult cardiomyocyte than that in fetal or neonatal counterpart. To demonstrate whether Yap activation can promote mature heart regeneration by stimulating adult cardiomyocyte proliferation, Xin et al. (2013) performed myocardial injury in wildtype and α*MHC-Yap^S112A^* transgenic mice at 4 weeks old and analyzed their hearts after 21 days. Similar to neonatal pups, adult α*MHC-Yap^S112A^* transgenic mice had improved regenerative response after myocardial injury when compared with their wildtype littermate controls (Xin et al., 2013). Similarly, Lin et al. (2014) described that cardiac-specific Yap activation in adult mouse heart stimulated cardiomyocyte proliferation, improved cardiac function, survival and reduced infarction size after myocardial injury.

Recently, Tao et al. (2016) observed that Pitx2 expression was induced in injured *Salv* knockout hearts. Conditional deletion of *Pitx2* in neonatal mouse heart resulted in impaired repair/regenerative response after apex resection, as evident by increased fibrosis and reduced cardiac function. However, *Pitx2* gain of function in adult cardiomyocytes facilitated cardiac repair and function (Tao et al., 2016). Genetic studies further revealed that Pitx2-Yap interaction is essential for maintaining the reparative capacity of adult cardiomyocytes after cardiac tissue injury (Tao et al., 2016).

Extracellular matrix composition also plays an important role in regulating the proliferation and differentiation of neonatal cardiomyocytes (Bassat et al., 2017). Genetic deletion causing changes in extracellular matrix composition can limit the neonatal mouse hearts’ regenerative capacity. For example, conditional deletion of Agrin (*Agrn*) in the heart leads to impaired cardiac regeneration in neonatal mice after apex resection, evident by increased fibrosis and reduced cardiomyocyte proliferation and cardiac function (Bassat et al., 2017). The study further demonstrated that Agrin interacts with its receptor α-Dystroglycan (Dag1) and promotes disassembly of the dystrophin-glycoprotein complex (DGC), leading to Yap translocation to the nucleus, where it promotes cardiomyocyte proliferation (Bassat et al., 2017). The DGC complex components such as dystrophin and Dag1 play an important role in mediating interactions among the cytoskeleton, membrane, and extracellular matrix. Disruption of the DGC complex leads to destabilization of the plasma membrane, making the cardiomyocytes more susceptible to stretch- or contraction-induced injury and cell death (Weller et al., 1990; Petrof et al., 1993; Dellorusso et al., 2001). Consistent with these findings, Morikawa et al. (2017) also reported that Yap interaction with Dag1 prevents its nuclear translocation and inhibits cardiomyocyte proliferation. This interaction was further characterized (Morikawa et al., 2017) in a *Mdx* knockout mouse model (dystrophin loss of function). Using the neonatal cardiac apex resections injury model at P8, the authors showed that cardiomyocyte-specific *Salv* mutant hearts regenerated efficiently with little or no tissue fibrosis, while the control and *Mdx* (dystrophin mutant) hearts were unable to regenerate (Morikawa et al., 2017). Interestingly, *Salv*;*Mdx* double-knockout hearts regenerated efficiently with excessive myocardial growth and less fibrosis, suggesting an improvement in cardiac regeneration (Morikawa et al., 2017). These findings suggest that either inhibition of Hippo-kinases or Yap activation could be a potential therapeutic strategy to improve survival and cardiac function after myocardial injury.

## The Hippo Signaling Pathway in Cardiac Diseases

Deregulation of the Hippo signaling pathway has been associated with various cardiac abnormalities including cardiac hypertrophy, heart failures, arrhythmogenic cardiomyopathy, dilated cardiomyopathy and ischemic heart disease (Tables 1, 2).

## Cardiac Hypertrophy

In the developing hearts, cardiomyocyte proliferation is mainly initiated by pre-existing cardiomyocytes. However, since adult cardiomyocytes have restricted proliferative capacity, postnatal heart growth is governed by the physiological hypertrophy of the cardiomyocytes (Ahuja et al., 2007). Cardiac hypertrophy is also induced by pathological conditions such as myocardial ischemia/reperfusion (I/R) injury and myocardial infarction (MI). These conditions result in oxidative stress and irreversible loss of cardiomyocytes leading to heart failure. Adaptation to these adverse conditions causes insufficient contractile force where the ventricular cardiomyocytes undergo hypertrophy to increase the wall thickness and reduce wall stress, as well as cell death (Frey et al., 2004; Tardiff, 2006; Samak et al., 2016). Several studies have demonstrated that Yap activation under physiological and pathological conditions increases cardiomyocyte proliferation in both fetal and postnatal hearts without affecting its size (Xin et al., 2011, 2013; von Gise et al., 2012). Similarly, Lin et al. (2014) also observed that Yap activation increases cardiomyocyte proliferation without affecting hypertrophy and protects the heart from myocardial infarction. In contrast, Sadoshima and colleagues demonstrated that Yap prevents myocardial infarction by promoting not only cardiomyocyte survival and proliferation but also hypertrophy (Del Re et al., 2013). They further showed that cardiomyocyte survival resulting from Yap activation is mediated by either AKT/miR-206 or both, while its effect on hypertrophy is solely mediated by miR-206 (Yang et al., 2015). A recent clinical study demonstrated that Yap expression was increased whereas Mst1 expression was significantly reduced in heart samples from patients with hypertrophic cardiomyopathy (Wang P. et al., 2014). Similarly, Yap expression was also increased in mice with transverse aortic constriction (TAC), suggesting its important role in hypertrophic heart disease. Importantly, inhibition of Yap phosphorylation at serine 127 was reduced in both human and mouse tissues (Wang P. et al., 2014). Moreover, cardiomyocyte-specific overexpression of human Yap induced both *in vitro* and *in vivo* hypertrophy (Wang P. et al., 2014). Consistent with these findings, both Yap’s pro-hypertrophic and pro-survival functions were activated during TAC-induced adoptive hypertrophic response (Byun et al., 2019).

Several upstream Hippo signaling kinases are also implicated in cardiac hypertrophy. Inhibition or deletion of upstream Hippo kinases induces nuclear accumulation of Yap. Mst1 is also activated in response to I/R injury, myocardial infarction, and pressure overload. Surprisingly, neither cardiac-specific overexpression of wildtype nor the dominant-negative form of Mst1 (suppresses endogenous Mst1) caused any significant changes in cardiac function or cardiac hypertrophy in mice at baseline conditions (Yamamoto et al., 2003; Odashima et al., 2007). To further explain the role of Mst1, Del re et al. reported that elevation of Ras-association domain family 1 isoform A (Rassf1A), a physiological activator of Mst1, is associated with cardiomyocyte apoptosis and cardiac hypertrophy (Oceandy et al., 2009; Del Re et al., 2010). Rassf1A expression in the mouse heart is initially increased at 1 week but decreased after TAC-induced hypertrophy at 12 weeks. Rassf1A expression is also decreased in failing human hearts (Oceandy et al., 2009; Del Re et al., 2010). Subsequently, cardiomyocyte-specific ablation of *Rassf1a* or overexpression of Mst1-binding-mutant form of Rassf1A reduced TAC-induced hypertrophy and fibrosis in mice (Del Re et al., 2010). In contrast to Mst1, *Mst2* knockout mice showed attenuated hypertrophy, while Mst2 overexpression increased hypertrophy in response to pressure overload (Zi et al., 2014). However, Mst2’s impact on hypertrophy was exerted through activation of the prohypertrophic Raf1-ERK1/2 pathway and not Yap (Zi et al., 2014). Similar to Mst kinases, Lats2 is also upregulated in response to pressure overload in mice (Matsui et al., 2008). Lats2 is also a negative regulator of cardiac hypertrophy (Matsui et al., 2008), where its overexpression did not affect cardiomyocyte apoptosis but rather reduced cardiomyocyte size. Cardiomyocyte-specific overexpression of a dominant-negative form of Lats2 notably increased ventricular hypertrophy and suppressed TAC-induced cardiomyocyte apoptosis (Matsui et al., 2008).

Sciarretta et al. (2015) recently demonstrated the cross-talk between mTOR and Hippo signaling pathway is essential for determining the cardiac response to pressure overload. The authors demonstrated that mTORC2, one of the multiprotein complexes mTOR forms with adaptor proteins, is a negative regulator of the Mst1 kinase, a key component of the Hippo signaling pathway (Sciarretta et al., 2015). mTORC2 reduces Mst1 homodimerization and its activity by phosphorylating specific serine residues in the SARAH domain. Rictor is the main components of the mTORC2 complex, where its deletion disrupts the whole complex (Wullschleger et al., 2006; Laplante and Sabatini, 2012). Sciarretta et al. (2015) showed that cardiac-specific mTORC2 disruption through *rictor* deletion in 6-month-old mice resulted in Mst1 activation, leading to dilation and impaired cardiac function. Increased cardiac fibrosis and apoptosis were also observed in *rictor* knockout mice, where they developed severe heart failure in response to pressure overload. In addition, compensatory hypertrophic response was also attenuated, suggesting that Rictor/mTORC2 regulates pressure overload-induced cardiac hypertrophy (Sciarretta et al., 2015). Collectively, Hippo-pathway plays an essential role in modulating the cardiac response to stress.

## Ischemic Heart and Dilated Cardiomyopathy

Ischemia-reperfusion injury is one of the most common causes of cardiac dysfunction in human. It is widely accepted that cardiomyocyte death after IR injury is largely due to excessive production of reactive oxygen species (ROS) and activation of cell death pathways. The role of Hippo signaling pathway in regulating cardiomyocyte apoptosis has been extensively studied. Several studies have reported that activation of Hippo-kinase Mst1 is associated with cardiomyocyte apoptosis and dilated cardiomyopathy (Yamamoto et al., 2003; Odashima et al., 2007; Del Re et al., 2010). Mst1 is activated *in vitro* by pro-apoptotic stimuli in cardiomyocytes causing apoptosis in a kinase activity-dependent manner. Furthermore, Mst1 is activated in cardiomyocytes due to oxidative stress induced by I/R injury in the mouse heart (Yamamoto et al., 2003). Cardiac-specific overexpression of *Mst1* in mice resulted in activated caspases, increased cardiomyocytes apoptosis and deterioration of cardiac function from heart dilation, all of which result in some premature death (Yamamoto et al., 2003). However, suppression of endogenous *Mst1* with cardiac-specific overexpression of a dominant-negative form of Mst1 (Mst1^K59R^) inhibited cardiomyocytes apoptosis, reduced the extent of myocardial damage and improved cardiac function after I/R injury or myocardial infarction (Yamamoto et al., 2003; Odashima et al., 2007).

A recent study reported elevated phosphorylated Yap and Lats protein levels in the heart samples from patients with ischemic or non-ischemic heart failures (non-ischemic idiopathic end-stage cardiomyopathy) (Hou et al., 2017; Leach et al., 2017). However, no change in Salv levels was observed. Compared with control littermates, cardiomyocyte-specific inducible deletion of *Salv* in mice reduced ischemia-induced fibrosis and improved cardiac function (Leach et al., 2017). Consistent with these results, direct myocardial or systemic injection of adeno-associated virus 9 (AAV9) encoding short hairpin RNA (shRNA) against Salv improved cardiomyocyte cell cycle re-entry and cardiac function after myocardial infarction (Leach et al., 2017).

Duchenne Muscular Dystrophy (DMD) is a disorder where progressive muscle weakness causes dilated cardiomyopathy resulting in heart failure and myocardial fibrosis. DMD is caused by loss-of-function mutations in the dystrophin gene. To demonstrate the role of Hippo signaling pathway in dilated cardiomyopathy, Morikawa et al. (2017) generated *Salv*;*Mdx* double knockout mice and analyzed them after TAC, an overload model of dilated cardiomyopathy (Kassiri et al., 2005). *Mdx* hearts developed TAC-induced dilated cardiomyopathy with loss of cardiomyocytes, increased fibrosis and impaired cardiac function. In contrast, inactivation of the Hippo pathway in *Mdx* genetic background (*Salv;Mdx* double knockout) protected the heart from TAC-induced dilated cardiomyopathy by increasing Yap activity, thereby increasing cardiomyocyte proliferation and decreasing apoptosis (Morikawa et al., 2017).

Neurofibromin 2 (NF2) is a GTPase-activating protein that regulates the RAS signaling pathway (Matsuda et al., 2016). It has been described to modulate Mst1 and Lats2-mediated cardiomyocyte apoptosis after I/R injury (Matsuda et al., 2016). In response to oxidative stress triggered by I/R injury, NF2 is activated in the cardiomyocytes/myocardium. Knockdown of *NF2* using siRNA inhibited Mst1-mediated apoptosis in cultured cardiomyocytes (Matsuda et al., 2016). Following I/R injury, cardiomyocyte-specific deletion of *NF2* also demonstrated cardioprotective effect with reduced infarction and improved cardiac function (Matsuda et al., 2016; Mia et al., 2016). They also observed Yap activation in NF2 deficient heart after I/R injury and genetic ablation of Yap in NF2-deficient heart abolished its cardioprotective effects, as evident by enhanced apoptosis and increased infarction. Collectively, the Hippo-pathway plays a crucial role in controlling apoptosis and growth of cardiomyocytes, thereby regulating the size of ventricles and overall cardiac functions in the adult mouse heart.

## Arrhythmogenic Cardiomyopathy

The Hippo-Yap pathway was also reported in the pathophysiology of arrhythmogenic cardiomyopathy (Chen et al., 2014). Arrhythmogenic cardiomyopathy is an inherited heart muscle disorder characterized by a progressive fibro-fatty replacement of the ventricular myocardium, ventricular arrhythmias and impaired ventricular systolic function leading to sudden cardiac death. Chen et al. (2014) demonstrated that the Hippo pathway is activated in the human heart and mouse models of arrhythmogenic cardiomyopathy. They showed that activation of the hippo pathway is due to changes at the intercalated disks, as shown by markedly reduced expression of several intercalated disks proteins such as JUP, PKP2, DSC2, DSG2, DSP, and GJA1 in human patients with advanced arrhythmogenic cardiomyopathy. These changes resulted in the reduced localization of activated protein kinase C-alpha (PKCα), which normally phosphorylates and inactivates NF2. Consequently, elevated expression of NF2 was observed in the heart samples from patients and preclinical mouse model with arrhythmogenic cardiomyopathy (Chen et al., 2014). Activation of NF2 leads to phosphorylation of Hippo components Mst1/2, Lats1/2, and Yap, suppressing the Yap-Tead target gene expression. Based on the reported crosstalk between Wnt/β-catenin and Hippo/Yap signaling pathways described above, the authors determined the status of the Wnt/β-catenin signaling in arrhythmogenic cardiomyopathy. Although the total β-catenin levels were unchanged, its localization to the intercalated disks was affected, leading to reduced Wnt/β-catenin transcriptional activity (Chen et al., 2014). Together, these results suggest that reduced Wnt/β-catenin and Hippo/Yap signaling are associated with enhanced adipogenesis in arrhythmogenic cardiomyopathy. Furthermore, enhanced adipogenesis was normalized in mice with *Lats1/2* knockdown and those with active Yap expression. This suggests that activation of hippo kinases promote adipogenesis in arrhythmogenic cardiomyopathy (Chen et al., 2014). Collectively, these results demonstrate that activation of the Hippo pathway is mediated through the intercalated disks and thus provide a novel mechanism for the pathogenesis of arrhythmogenic cardiomyopathy.

## Post-Myocardial Infarction (MI) Inflammation and Remodeling

Many preclinical studies have established the role of Hippo pathway members in cardiac development, regeneration, and homeostasis. Recent advances suggest that the Hippo signaling components are also critically involved in regulating inflammatory processes, including the immune response. For example, Yap mediates recruitment of tumor-associated macrophages required for immune evasion and tumor progression (Guo et al., 2017; Huang et al., 2017). Likewise, Hagenbeek et al. (2018) reported that both Yap and Taz play a diverse role in liver tumor development where Taz stimulated proinflammatory cytokine production and macrophage infiltration, while Yap promoted tumor formation. It is also evident that Yap plays a vital role in regulatory T cells (Treg) mediated suppression of antitumor immunity (Ni et al., 2018). Despite the well-established role of Hippo signaling components in regulating the inflammatory response in different pathophysiological conditions, their role in modulating post-MI inflammation and remodeling has not been fully investigated.

A growing number of studies demonstrated that epicardium is reactivated after myocardial infarction. Reactivated epicardial cells express genes that are normally expressed in the embryonic epicardium. These cells also proliferate and cover the damaged tissue. A subset of activated epicardial cells undergo EMT and differentiate into fibroblasts and smooth muscle cells, which further contribute to the repair process. Epicardium also secretes angiogenic and inflammatory factors in response to cardiac injury, which subsequently increases inflammatory cell influx to the infarcted area. Recently, Ramjee at al. (2017) reported that Hippo signaling in the epicardium plays a crucial role in the myocardial recovery after injury by recruiting regulatory T (Treg) cells, a subset of CD4+ T cells, to the injured myocardium. Treg cells have been shown to decrease the immune response and infarct scar size in the myocardium after injury (Hofmann et al., 2012; Nahrendorf and Swirski, 2014; Weirather et al., 2014). Ramjee et al. (2017) demonstrated that epicardial-specific deletion of Yap and Taz leads to profound post-injury pericardial inflammation and ventricular fibrosis due to decreased number of Treg cells in the injured myocardium, resulting in cardiomyopathy and increasing mortality. Yap/Taz also regulates IFN-γ expression in the activated epicardium. Loss of Yap/Taz in the epicardium resulted in downregulation of IFN-γ. Furthermore, exogenous administration of IFN-γ using hydrogel improved Treg cells infiltration into the injured myocardium and decreased ventricular fibrosis (Ramjee et al., 2017). Similarly, Yap activation through TLR3 signaling is essential for neonatal heart regeneration after MI (Wang et al., 2018). Although the role of Hippo signaling in non-immune cells associated inflammatory response is extensively studied, its functions in immune cells are not well established (Perez-Lopez et al., 2013; Ni et al., 2018). A recent study demonstrated that Yap deletion in the myeloid lineage protected the mice from inflammatory bowel disease (Zhou et al., 2019). These suggest that Hippo pathway components may play a significant role in inflammatory cells by modulating the post-MI inflammatory response, controlling adverse remodeling and facilitating cardiac function recovery after MI.

## Conclusion

The Hippo signaling pathway has emerged as an essential regulator of cardiac development, growth and regeneration/repair after injury. In an animal model, deletion of Hippo-component Salv or activation of Yap improved cardiac function and survival after myocardial infarction (Lin et al., 2014; Leach et al., 2017). This suggests that Hippo signaling pathway can potentially be used as a target to improve cardiac regeneration/repair after injury. Although Yap activation appears to have short-term beneficial effects, its long-term consequences on the Hippo signaling pathway and cardiac injury remain to be elucidated. Some findings suggest that long-term Yap activation may have adverse effects on the heart (Wang P. et al., 2014; Monroe et al., 2019). This suggests that Yap activation in cardiomyocytes need to be tightly controlled to get the beneficial effects that improve the cardiac function after injury without major adverse effects. Recently, Hara et al. identified a compound that increased Yap-Tead activity and protected the heart against ischemic injury in mice (Hara et al., 2018). Although the Hippo signaling pathway has been extensively investigated in cardiomyocytes, its role in cardiac fibroblast and immune cells such as macrophages and T cells has not been well described. Since these cells play an important role in the repair process, further research is necessary to better understand how the Hippo signaling pathway regulates them in developing therapeutic interventions without potential adverse effects.

## Author Contributions

MM wrote the initial draft of the manuscript. MS wrote and edited the final manuscript.

## Conflict of Interest

The authors declare that the research was conducted in the absence of any commercial or financial relationships that could be construed as a potential conflict of interest.
